# The influence of teacher support on vocational college students’ information literacy: The mediating role of network perceived usefulness and information and communication technology self-efficacy

**DOI:** 10.3389/fpsyg.2022.1032791

**Published:** 2022-10-26

**Authors:** Qiaoyun Chen, Ying Ma

**Affiliations:** ^1^School of Educational Sciences, Nanjing Normal University, Nanjing, Jiangsu, China; ^2^Dushu Lake School in Suzhou Industrial Park, Suzhou, Jiangsu, China

**Keywords:** teacher support, higher vocational students, information literacy, usefulness of network perception, ICT self-efficacy

## Abstract

This paper uses the network perceived usefulness scale, Information and Communication Technology (ICT) self-efficacy scale, teacher support questionnaire and higher vocational students’ information literacy scale to explore the multiple intermediary functions of network perceived usefulness and ICT self-efficacy in teacher support and higher vocational students’ information literacy from the perspective of multiple intermediary effects, and uses structural equation model for data modeling and analysis. The results show that the information literacy of higher vocational students is positively correlated with teacher support, usefulness of network perception and ICT self-efficacy. Teachers’ support is positively correlated with ICT self-efficacy and network perceived usefulness, and network perceived usefulness and ICT self-efficacy play a significant mediating role between teachers’ support and information literacy of higher vocational students. Based on the research results, some suggestions are put forward to guide teachers to help students clearly recognize the role of information technology, improve ICT self-efficacy, improve information literacy and better integrate into digital society.

## Introduction

### Research background

At the information literacy conference in Prague in 2003, the United Nations Educational, Scientific and Cultural Organization (UNESCO) put forward that information literacy is a basic human right of lifelong learning and a prerequisite for individuals to join in the information society (Sun and Zeng, 2005). In 2015, “American Higher Education Information Literacy Competency Standards” clearly pointed out that information literacy is a part of the comprehensive quality evaluation of talents (Qin, 2015). Germany launched the “Framework of Vocational Education 4.0” in 2016, and put forward the vision of vocational education to cultivate professional qualifications and abilities for future digital work (Tian, 2021). In 2017, the Ministry of Education, Culture, Sports, Science and Technology of Japan (2022) proposed to deal with the impact of artificial intelligence (AI), big data and other technologies on the society by implementing the “Super Vocational High School Plan,” and in 2021, it issued the “Digital Initiative of University Education” to cultivate talents who can adapt to the “Industry 4.0” era ^(4)^. In 2021, China deployed the task of implementing the comprehensive improvement of information literacy, and proposed to build a new talent training model under the condition of “internet plus” ^(5)^. Therefore, the knowledge and skills of digital information technology with computer as the core have become the basic requirements of all social departments for the quality of talents. As applied and skilled reserve talents for social production, higher vocational students will surely become the main force in the upgrading and transformation of industrial structure in the future, and the cultivation of their information literacy has attracted the attention of governments all over the world.

### Research question

Previous studies have shown that that the information literacy level of vocational college students is not ideal (Li, 2021; Qin, 2021), and there are still many problems in information awareness, information acquisition, and information processing ability. Therefore, it is necessary to analyze the influencing factors and mechanism of information literacy of higher vocational students. The purpose of this study is to pay attention to how teachers’ learning support, emotional support and instrumental support affect students’ information literacy, and to analyze the action path and mechanism of the two factors, the usefulness of network perception and ICT (Information and Communication Technology) self-efficacy, between students’ information literacy and teachers’ support, so as to provide valuable theoretical and practical basis for the cultivation of students’ information literacy in higher vocational colleges.

## Literature review and hypothesis

### Teacher support and students’ information literacy

According to the ecosystem theory, the school is the micro-system that has the closest influence on students’ development besides the family environment (Eccles and Roeser, 2011), and teachers, as the key factor in this system, have the greatest influence on students (Hattie, 2005; Bayat et al., 2014). Teachers’ supportive behaviors to students in study or life can significantly affect students’ deep learning (Li, 2022). The more teachers’ support, the more positive experiences students have (Hao et al., 2021). There is a significant positive correlation between teachers’ perceived support and their academic performance (Zhang et al., 2019), and teachers’ supportive behavior can effectively predict students’ academic performance (Ji and Zhao, 2021).

In the fields of mathematics, reading and science, teacher support has become an important predictor of students’ literacy (Saroughi and Cheema, 2022), and it influences students’ reading literacy through the double intermediary of reading self-concept and reading enjoyment (Ma et al., 2020). Teachers’ support for cross-curriculum cooperation and professional development can promote students’ overall literacy, including information technology literacy (Brian, 2018). However, a survey of college students shows that more than 50% of them have low information literacy, which can’t meet their learning needs at school. Without the support of teachers, students often don’t take the time to learn information technology by themselves (Lanning and Mallek, 2017). Teachers’ attitude and interest in information technology is an important factor that affects students’ attitude toward learning information technology (Meelissen and Drent, 2008). In addition, teachers’ use of ICT information tools in teaching will affect students’ ICT ability, and teachers’ support is an important factor in students’ information literacy skills training (Aesaert et al., 2015). However, teacher support does not directly affect students’ ICT literacy, and other variables play an intermediary role (Kim et al., 2014).

Thus, there is a positive correlation between teacher support and learners’ information technology literacy, that is, teacher support can help learners better acquire information awareness and information ability.

On the basis of this analysis, we proposed the following hypotheses.

H1: Teacher support is significantly related to information literacy of higher vocational students.

### The mediating role of network perceived usefulness between teacher support and students’ information literacy

In the Technology Acceptance Model, Perceived Usefulness refers to the degree to which learners think that using a new technology may improve their academic performance, which directly affects the behavior intention of ordinary learners (Davis, 1989) and belongs to the influencing factor of students’ information literacy (Heerwegh et al., 2016). The analysis based on Item Response Theory model shows that students’ information literacy is positively correlated with their perceived usefulness of communication technology (Siddiq et al., 2017). Perceived usefulness has a significant positive impact on users’ online information search willingness and online information search behavior. For example, perceived usefulness will have a positive impact on the length and frequency of mobile reading of college students (Liu, 2021), and the online teaching usefulness of normal students will have a positive impact on their online teaching willingness (Liu et al., 2021).

The support of teachers and instructors has a significant impact on students’ perceived usefulness (Shen et al., 2006). Teachers’ support affects students’ learning behavior intention through the intermediary variable of perceived usefulness (Jiang and Jing, 2021), and there is a direct and positive relationship between teachers’ support and students’ perceived usefulness (Li et al., 2020).

It can be seen that perceived usefulness has a positive impact on students’ information awareness and information behavior (an important part of information literacy), while teacher support has an impact on students’ information literacy through the intermediary variable of perceived usefulness.

Based on the above analysis, we proposed the following hypotheses.

H2: Teacher support has a significant positive impact on the perceived usefulness of the network.

H3: The usefulness of network perception has a significant positive impact on the information literacy of higher vocational students.

H4: The usefulness of network perception plays an intermediary role between teacher support and information literacy of higher vocational students.

### Information and communication technology self-efficacy plays an intermediary role between teacher support and vocational students’ information literacy

Information and communication technology self-efficacy is the extension and development of Bandura’s self-efficacy theory in ICT environment, which refers to the degree to which individuals believe that they can accomplish ICT-related tasks. Research on a large number of students’ ICT literacy proves that there is a significant positive correlation between students’ ICT literacy and self-efficacy (Siddiq et al., 2017). Students’ ICT self-efficacy has a significant positive impact on their information literacy (Hatlevik et al., 2015; Scherer et al., 2017). Therefore, a common way to measure digital skills (including information literacy) is to ask people to assess their general abilities and attitudes toward information and communication technologies, such as self-efficacy related to information and communication technologies (Zhong, 2011). Students’ computer self-efficacy is an important predictor of their digital ability (literacy).

There is a significant positive correlation between teachers’ support for students and their academic self-efficacy or self-efficacy (Ji and Zhao, 2021). It will indirectly affect students’ learning input through academic self-efficacy. Studies have found that students’ ICT experience, usage and attitude will affect their information literacy, and students’ motivation beliefs (including self-efficacy) can be changed by teachers’ teaching and other support and intervention (Hatlevik et al., 2015). The full use of ICT by school teachers to support learning, teaching and management will help learners take responsibility for their own learning and build self-awareness and confidence (Tarus et al., 2015). Teachers’ support and intervention have a positive impact on students’ confidence in using ICT skills (Wang et al., 2014).

Therefore, we believe that teacher support can indirectly affect learners’ information literacy through ICT self-efficacy.

Based on the above analysis, we proposed the following hypotheses.

H5: Teacher support has a significant positive impact on ICT self-efficacy.

H6: ICT self-efficacy has a significant positive impact on vocational college students’ information literacy.

H7: ICT self-efficacy plays an intermediary role between teacher support and information literacy of higher vocational students.

**FIGURE 1:**
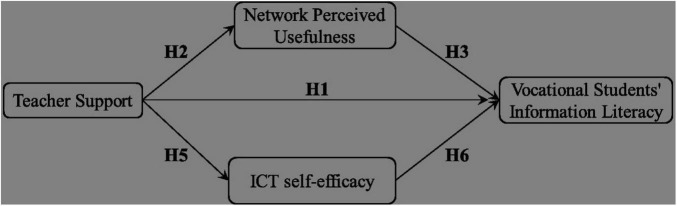
Theoretical model of the relationship between teacher support and information literacy of higher vocational students.

The theoretical model of the influence of teacher support on vocational college students’ information literacy constructed in this study is shown (Figure 1).

## Methodology

### Data sources

In this study, 455 students from higher vocational colleges in Jiangsu province in eastern China were investigated. By stratified random sampling, 10 colleges were randomly selected from 90 higher vocational colleges in Jiangsu province, and all three grades of each college were selected as the survey objects. A total of 455 questionnaires were distributed, and 449 valid questionnaires were obtained, with a recovery rate of 98.7%. The subjects included 96 male (21.38%) and 353 female (78.62%). A total of 137 (24.95%) in Grade One, 261 (58.13%) in Grade Two and 51 (11.36%) in Grade Three; there are 183 urban students (40.76%) and 266 rural students (59.24%).

### Research tools

The questionnaire mainly includes four scales, namely, Teacher Support Scale, Network Perceived Usefulness Scale, ICT Self-efficacy Scale, and Student Information Literacy Scale. All the items in the scale are scored by Likert’s five-point scale, with 1 indicating complete disagreement and 5 indicating complete agreement.

### Teacher support scale

Teacher support scale is used to test students’ perceived teacher support. The teacher support questionnaire (Ouyang, 2005) compiled by Ouyang Dan is used, which includes 19 questions in three dimensions: teacher learning support, teacher emotional support and teacher instrumental support. For all items, the higher the score, the more teachers’ support students perceive. Cronbach’s α coefficient of the questionnaire is 0.87, and Cronbach’s α coefficient of each dimension is 0.84, 0.73, and 0.79, respectively.

#### Network perceived usefulness scale

The network perceived usefulness scale uses Wei’s (2018) scale adapted from Cai Jinzhong’s achievements, and has five questions. The higher the score of the questions, the higher the students’ network perceived usefulness. The rotation factor load of each topic of the scale is greater than 0.6, which indicates that the scale has good validity. Cronbach’s α coefficient is 0.90, which indicates that the scale has good reliability.

#### Information and communication technology self-efficacy scale

Information and communication technology Self-efficacy Scale uses the ICT Self-efficacy Scale compiled by Musharraf et al. (2019) which includes three dimensions: privacy and security, differentiation and learning, and communication. There are 18 questions in total. The higher the score of the topic, the higher the usefulness of students’ network perception and ICT self-efficacy. The reliability and validity of the scale are 0.93 and 0.92, respectively; the reliability and validity of the three dimensions are 0.89 and 0.93, 0.81 and 0.83, 0.67 and 0.67 respectively.

#### Information literacy scale for higher vocational students

The information literacy scale for higher vocational students is based on the Information Literacy Ability Standard of Higher Education of American Association of Universities and Research Libraries and the Information Literacy Evaluation Scale for Higher Vocational Students in China (Mo, 2007), which includes 50 topics in four dimensions: information awareness, information knowledge, information ability and information morality. The topics are divided into five levels. The higher the score, the higher the information literacy level of students. The KMO value of the questionnaire is 0.963, the Cronbach’s α coefficient of the total scale is 0.942, and the α coefficients of the subscales of information consciousness, information morality, information knowledge and information ability are 0.652, 0.783, 0.763, and 0.927, respectively. The reliability and validity of the questionnaire are all good.

### Data processing

In this study, SPSS 22.0 is used to analyze the reliability and validity of the questionnaire to ensure the scientificity and availability of the collected data, and AMOS 21.0 is used to construct the structural equation model, estimate the fitting degree of the whole model, and analyze the relationship among variables to verify whether there is an intermediary effect.

## Results

### Gender differences in teacher support, perceived usefulness of network, information and communication technology self-efficacy, and information literacy

The difference analysis results show that there are significant differences in teachers’ support, ICT self-efficacy and overall scores of students’ information literacy scale among vocational college students of different genders, among which male students consider that teachers’ support (*p* = 0.010 < 0.05), ICT self-efficacy (*p* = 0.001 < 0.05), and information literacy (*p* = 0.025 < 0.05) are significantly higher than female students. There is no significant difference between male and female students in perceived usefulness of network (*p* = 0.120 > 0.05).

### Grade differences in teacher support, perceived usefulness of network, information and communication technology self-efficacy, and information literacy

There are no significant differences in teachers’ support, ICT self-efficacy, usefulness of network perception and information literacy between grade 1 and grade 2 and grade 3 students (see Table 1).

**TABLE 1:** Analysis results in grade differences.

Score of	Grade 1 (*N* = 137) M ± SD	Grade 2 (*N* = 261) M ± SD	Grade 3 (*N* = 51) M ± SD	P (grade 1 and grade 2)	P (grade 2 and grade 3)	P (grade 1 and grade 3)
Teacher support	74.42 ± 12.14	72.70 ± 12.20	72.76 ± 12.44	0.180	0.971	0.409
Usefulness of network perception	21.79 ± 3.30	21.33 ± 3.41	21.16 ± 3.55	0.198	0.743	0.254
ICT self-efficacy	67.48 ± 13.28	67.92 ± 13.17	69.47 ± 13.22	0.754	0.443	0.362
Information literacy	200.43 ± 33.37	196.56 ± 35.16	204.57 ± 36.16	0.288	0.139	0.461

### Correlation analysis of teacher support, perceived usefulness of network, information and communication technology self-efficacy, and information literacy

Descriptive statistical analysis and correlation analysis are made on the variables of teacher support, network perceived usefulness, ICT self-efficacy and information literacy (Table 2). Among them, the total score of teacher support scale is 95, the total score of network perceived usefulness scale is 25, the total score of ICT self-efficacy scale is 90, and the total score of information literacy scale of higher vocational students is 250. Higher vocational students’ information literacy is positively correlated with teacher support, usefulness of network perception and ICT self-efficacy. Teacher support is positively correlated with ICT self-efficacy and perceived usefulness of the network. Therefore, it can be judged that the higher the teachers’ support level that students in higher vocational colleges can perceive, the higher their usefulness of network perception and ICT self-efficacy, and the higher their self-perceived information literacy level.

**TABLE 2:** Correlation analysis results of teacher support, usefulness of network perception, ICT self-efficacy and information literacy of higher vocational students.

Variable	1	2	3	4
1. Teacher support	1			
2. Usefulness of network perception	0.650**	1		
3. ICT self-efficacy	0.582**	0.567**	1	
4. Information literacy	0.640**	0.693**	0.761**	1
Average	73.23	21.45	67.96	198.65
Standard deviation	12.207	3.394	13.192	34.766

**p < 0.01.

### Independent influence of teacher support on vocational students’ information literacy

This study adopts hierarchical regression method, taking teacher support as independent variable and gender, grade and students’ place of origin as control variables, and establishes a model to explore the independent influence of teacher support on higher vocational students’ information literacy. The results show that, in the absence of control variables (model 1), teacher support has a significant positive correlation with vocational college students’ information literacy, and its explanatory power is 40.8%. After the control variables are put into the model (model 2), there is still a significant positive correlation between teacher support and vocational college students’ information literacy, and the explanatory power of teacher support to vocational college students’ information literacy is slightly improved, reaching 41.0% (Table 3).

**TABLE 3:** Independent influence of teacher support on vocational students’ information literacy.

	Higher vocational students’			
Variable	information literacy			
	
	1 model		2 model	
		
	β (S.E.)	*t*	β (S.E.)	*t*
Teacher support	0.640 (0.104)	17.595***	0.632 (0.105)	17.128***
Model *R*^2^ (adjusting *R*^2^)	0.409 (0.408)		0.410 (0.410)	

***p < 0.001.

In order to understand the independent influence of each variable of teacher support on the information literacy of higher vocational students, this paper explores the independent influence of teacher learning support, teacher emotional support and teacher instrumental support on the information literacy of higher vocational students by regression method. The results show that teachers’ learning support, teachers’ emotional support and teachers’ instrumental support are positively correlated with higher vocational students’ information literacy. Among them, teachers’ learning support alone accounts for 36.3%, teachers’ emotional support alone accounts for 37.8%, and teachers’ instrumental support alone accounts for 25.2% (Table 4). It can be seen that teachers’ learning support has the greatest influence on the cultivation of higher vocational students’ information literacy, followed by emotional support.

**TABLE 4:** Independent influence of different factors of teacher support on information literacy of higher vocational students.

Variables and factors		Higher vocational students’ information literacy		
		
		β (S.E.)	*t*	Model *R*^2^ (adjusting *R*^2^)
Teacher support	Teacher learning support	0.604 (0.220)	16.017***	0.365 (0.363)
	Teachers’ emotional support	0.616 (0.317)	16.526***	0.379 (0.378)
	Teachers’ instrumental support	0.504 (0.424)	12.323***	0.254 (0.252)

***p < 0.001.

### The mediating role of network perceived usefulness and information and communication technology self-efficacy

Previous studies have shown that teacher support affects learners’ network perceived usefulness and ICT self-efficacy, which can significantly predict students’ information literacy. Based on this, we have constructed a multiple mediation model to test the mediation effect of network perceived usefulness and ICT self-efficacy between teacher support and higher vocational students’ information literacy.

**FIGURE 2:**
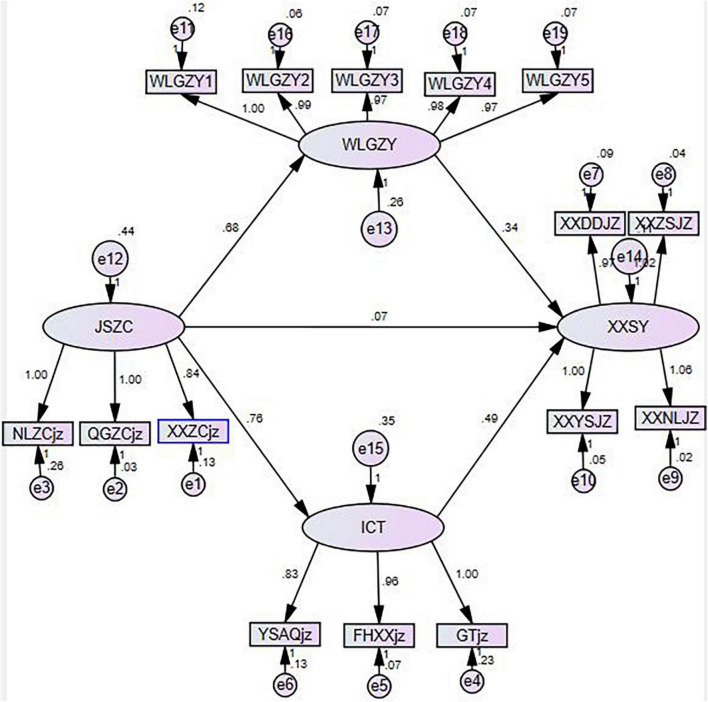
Mediating model diagram of network perceived usefulness and ICT self-efficacy.

This paper analyzes the relationship among teacher support, usefulness of network perception, ICT self-efficacy and information literacy of higher vocational students by structural equation model. Taking teacher’s support as the predictive variable, information literacy of higher vocational students as the outcome variable, and network perceived usefulness and ICT self-efficacy as the intermediary variables, the fitting index of the model is X2/df = 4.657, NFI = 0.926; CFI = 0.937; GFI = 0.901; RMSEA = 0.071. Figure 2 shows that teacher support can significantly positively predict the usefulness of network perception (path coefficient is 0.68, *P* < 0.001) and ICT self-efficacy (path coefficient is 0.76, *P* < 0.001), but it cannot directly predict the information literacy of higher vocational students (path coefficient is 0.07, *P* = 0.105 > 0.05). The perceived usefulness of the network significantly positively predicts the information literacy of higher vocational students (path coefficient is 0.34, *P* < 0.001); ICT self-efficacy can significantly and positively predict the information literacy of higher vocational students (path coefficient is 0.49, *P* < 0.001). The results show that the path coefficients of network perceived usefulness and ICT self-efficacy are significant, but the path coefficients of the direct impact of teacher support on higher vocational students are not significant.

To test the mediating effect between teacher support and vocational college students’ information literacy, this study adopted the non-parametric percentage Bootstrap test with deviation correction, repeated sampling for 2,000 times, and calculated the 95% confidence interval, thus illustrating the significance of the mediating effect. The statistical results are shown in Table 5. The confidence interval of indirect effect of teacher support → perceived usefulness of network → information literacy of higher vocational students is [0.167, 0.309], and that of indirect effect of teacher support → ICT self-efficacy → information literacy of higher vocational students is [0.272, 0.498]. Both confidence intervals do not include 0, which indicates that the intermediary effect is significant. The confidence interval of the direct effect of teacher support → higher vocational students’ information literacy is [−0.49, 0.185], including 0, indicating that the direct effect is not significant.

**TABLE 5:** The test of mediating effect of network perceived usefulness and ICT self-efficacy between teacher support and information literacy of higher vocational students.

Effect	Path relation	Effect value	95% confidence interval	Effect quantity
Direct effect	Teacher Support → Information Literacy of Higher Vocational Students	0.069	[−0.49, 0.185]	10.30%
Mesomeric effect	Teacher support → Perceived usefulness of network → Information literacy of higher vocational students	0.028	[0.167, 0.309]	34.03%
	Teacher Support → ICT Self-efficacy → Information Literacy of Higher Vocational Students	0.373	[0.272, 0.498]	55.67%
Total effect		0.67	[0.57, 0.781]	

## Discussion

There are significant differences between male and female vocational college students in teacher support, ICT self-efficacy and students’ information literacy. Among them, the self-esteem of male students is significantly higher than that of female students. This is similar to the research results of Dolenc and Sorgo (2020) on the information literacy of Slovenian junior high school students, and man students generally have higher evaluation on their own abilities. This result is different from Ngo and Walton’s (2016) assessment of information literacy level of Vietnamese high school students, and also different from Fabbi’s (2012) prediction of ICT literacy level of Freshmen in the University of Nevada. The former survey shows that the information literacy level of female students is higher than that of male students, while the latter shows that there is no significant difference between male and female students. Such survey results may reflect that the differences between male and female students in country, region, grade and other aspects will lead to differences in their information literacy self-evaluation. In this study, there is no significant difference between male and female in the perceived usefulness of the network, which is also confirmed to a certain extent.

There were no significant differences in teacher support, ICT self-efficacy, network perceived usefulness and information literacy questionnaire scores among higher vocational students of different grades. This is similar to the results of Wu (2021) survey on Information Literacy of secondary vocational school students in Guangdong Province, which shows that among the five dimensions of information literacy, only the information attitude and consciousness dimension have some grade differences. This may be because the age difference of the students in the three grades is small, and the school environment, society and teachers’ strength that they contact are not different in informatization, so it is difficult to produce significant differences among the three grades.

Among the three dimensions of teachers’ support, teachers’ emotional support has the highest explanatory power to vocational students’ information literacy, teachers’ learning support is slightly lower, and teachers’ social support is the lowest. This means that teachers’ emotional support and learning support can positively affect the information literacy of higher vocational students, and the two kinds of support are roughly the same, which may be related to the close relationship between the two support methods. Generally speaking, students who receive more care and love from teachers are more likely to get help and guidance from teachers in learning. It shows that as educators and promoters of students, teachers have a subtle influence on students’ learning and comprehensive quality.

The results of this study show that information literacy of higher vocational students is significantly positively correlated with teacher support, network perceived usefulness and ICT self-efficacy, and teacher support is also significantly positively correlated with ICT self-efficacy and network perceived usefulness. This is consistent with the research results in Aesaert et al. (2015), Yang et al. (2018), and Zhang et al. (2019), which verifies the hypothesis that H2, H3, H5, and H6 can be established.

Through the calculation of the structural equation model constructed in this paper, the results show that the path coefficients with network perceived usefulness and ICT self-efficacy as intermediary variables are significant, but the path coefficients of the direct impact of teacher support on higher vocational students are not significant. This verifies that hypothesis H4 and H7 are valid but hypothesis H1 is not. This is the result obtained through investigation and structural equation model calculation in this paper, which is consistent with the conclusion of the study of Kim et al. (2014), but different from many research results. This may mean that among the factors that affect the information literacy of higher vocational students, teacher support is the external cause, while the perceived usefulness of the network and ICT self-efficacy are the internal causes, and the external factors play a role through the internal causes.

## Conclusion

In this study, the questionnaire method was used to collect data, and the structural equation model method was used to analyze the data. The interaction among teacher support, network perceived usefulness, ICT self-efficacy and information literacy of higher vocational students was observed. Therefore, the following conclusions are drawn from the results of empirical analysis.

### Gender differences in teachers’ support, information and communication technology self-efficacy and information literacy

Through the difference analysis, it can be seen that there are significant differences in the overall scores of teachers’ support, ICT self-efficacy and information literacy among vocational college students of different genders, among which male students’ teachers’ support, ICT self-efficacy and information literacy are significantly higher than female students’. Compared with female students, male students have more contact with the Internet, and their ICT self-efficacy and information literacy are relatively high.

### No grade differences in teachers’ support, information and communication technology self-efficacy, network perceived usefulness, and information literacy

Through the difference analysis, it can be seen that there are no significant differences in teachers’ support, ICT self-efficacy, the usefulness of network perception and the overall scores of information literacy questionnaire among higher vocational students of different grades. There is little difference in the age of the students in the three grades, and there is little difference in the information of the school environment, society and teachers. Therefore, there is no significant difference in the overall scores of teachers’ support, ICT self-efficacy, usefulness of network perception and information literacy questionnaire among the three grades of higher vocational students.

### Significant impact of teacher support on information literacy

Through correlation analysis, it can be seen that the information literacy of higher vocational students is positively correlated with teacher support, usefulness of network perception and ICT self-efficacy. Teacher support is positively correlated with ICT self-efficacy and perceived usefulness of the network. According to the data analysis of the independent influence of teacher support on vocational college students’ information literacy, it can be seen that teacher support has a significant positive correlation with vocational college students’ information literacy, and its explanatory power is 40.8%.

Further analysis shows that the three dimensions of teacher support, namely, teacher’s learning support, teacher’s emotional support and teacher’s instrumental support, have different influences on higher vocational students’ information literacy. Among them, teachers’ learning support has the highest explanatory power on information literacy of higher vocational students, reaching 36.3%; the sole explanatory power of teachers’ emotional support to higher vocational students’ information literacy comes second, reaching 37.8%; Teachers’ instrumental support has the smallest explanatory power on vocational college students’ information literacy, accounting for 25.2%.

### Significant mediating role of network perception usefulness and information and communication technology self-efficacy between teacher support and information literacy

Bootstrap analysis data shows that two mediating variables in the multiple mediating model play a significant mediating role between teacher support and information literacy of higher vocational students; with the participation of multiple intermediary effects, the direct effect of teacher support on higher vocational students’ information literacy is still significant. As mentioned above, only 10.30% of the effects of teacher support on vocational college students’ information literacy are direct, and the remaining 90.70% are mediated by two variables: perceived usefulness of network and ICT self-efficacy. Among them, 34.03% perceived usefulness through network, and 55.67% perceived usefulness through ICT.

It can be concluded that the usefulness of network perception and ICT self-efficacy are two important intermediary factors in the process of influencing vocational college students’ information literacy through teacher support. The clearer the higher vocational students’ cognition of teacher support and its value in education, the greater the intermediary role; The higher the level of students’ network perceived usefulness and ICT self-efficacy, the greater the mediating effect.

### Suggestions

Through the research, it is found that male students’ teachers’ support level, ICT self-efficacy and information literacy level are significantly higher than female students’, so it is necessary to strengthen female students’ information literacy, improve their ICT self-efficacy and improve their information literacy.

The three dimensions of teacher support influence the information literacy of higher vocational students in descending order: teacher’s learning support, teacher’s emotional support and teacher’s instrumental support. Among them, teachers’ instrumental support has the lowest influence on students, which is also related to teachers’ inability to provide students with enough instrumental support. Teachers play a leading role in teaching, so they must improve their knowledge structure and information literacy in time. If teachers can’t adjust their teaching strategies and methods in time to adapt to the information age, the function of information education will be restrained and restrained by the traditional teaching mode. Therefore, establishing a high-quality teaching staff in higher vocational colleges is the key to cultivate and improve the information literacy of students in higher vocational colleges.

In the process of influencing vocational college students’ information literacy through teacher support, the usefulness of network perception and ICT self-efficacy are two important intermediary factors. The clearer the higher vocational students’ cognition of teacher support and its value in education, the greater the intermediary role; The higher the level of students’ network perceived usefulness and ICT self-efficacy, the greater the mediating effect. However, the level of network perceived usefulness and ICT self-efficacy of higher vocational students is not high, so teachers should consciously cultivate students’ network perceived usefulness and ICT self-efficacy in ordinary teaching, so as to promote the improvement of information literacy.

## Data availability statement

The raw data supporting the conclusions of this article will be made available by the authors, without undue reservation.

## Author contributions

QC participated in conceptualization, supervision, design of experimental methods, practical investigation analysis of experimental data, revising of the manuscript, and was also responsible for the specific communication, manuscript revision, submission, and publication. YM participated in the actual investigation, experimental data analysis, experimental results visualization, and writing of the first draft of the manuscript. Both authors contributed to the article and approved the submitted version.
