# Supplementary material for: The influence of teacher support on vocational college students’ information literacy: The mediating role of network perceived usefulness and information and communication technology self-efficacy
Source: Front Psychol. 2022 Oct 26;13:1032791. doi: 10.3389/fpsyg.2022.1032791 (PMC9643587; doi:10.3389/fpsyg.2022.1032791)
Supplement: Supplementary file 1 [file Table_1.DOCX]

Questionnaire(in Chinese)

Dear students

Hello! Thank you very much for taking precious time out of your busy schedule to participate in this questionnaire survey!

This survey is conducted anonymously, mainly to understand your relevant situation in your ordinary learning process. The information you fill in is only for academic research, please answer carefully.

This questionnaire consists of five parts, all of which are multiple choice questions. In the process of filling in the questionnaire, please read the questions carefully and fill them in according to the actual situation or feelings. Please do not omit or make mistakes. Thank you for your warm support and patience!

**Part I: your basic information**

Your gender: A male B female

Your grade:

Your place of origin: A town; B countryside

Your major:

**Part II: teacher support**

The following table is used to evaluate the support you feel from teachers (19 questions). Please choose the one that best suits you for each of the following statements according to your situation.

1 = totally disagree, 2 = not agree, 3 = relatively agree, 4 = mostly agree, 5 = totally agree

1. In my study and life, the teacher is very strict with me

2. The teacher often asks me to take charge of the class affairs

3. The teacher has always been very gentle to me

4. When I answer a question, whether it's right or wrong, the teacher will tell me

5. Teachers often recommend me to participate in various activities or competitions

6. When I do well in class, the teacher will praise me

7. The teacher would signal me to get up and answer questions with encouraging eyes

8. The teacher thinks that I always have the ability to complete difficult homework or tasks

9. When I answer questions, the teacher will look me in the eye

10. My homework is often praised by the teacher

11. When I can't answer questions or make mistakes in class, the teacher always criticizes me

12. My teacher has always supported me to participate in various activities and competitions

13. In class, the teacher often asks me to answer questions

14. When I can't answer the question, the teacher often explains the question repeatedly to me

15. When I answer questions, the teacher will look at me with a smile

16. Teachers often encourage me in my study and life

17. I often feel that my teachers have high expectations of me

18. When I make mistakes in answering questions, the teacher will explain to me the reasons for the mistakes and how to correct them

19. When I can't answer a question, the teacher will often give me hints until I can answer it

**Part III: the usefulness of network awareness**

The following table is a self-assessment scale (5 questions) used to evaluate the usefulness of network perception. Please choose the one that best suits you for each of the following statements according to your situation.

1 = totally disagree, 2 = not agree, 3 = relatively agree, 4 = mostly agree, 5 = totally agree

1. The Internet can let me do more interesting and imaginative things

2. The Internet can broaden my vision

The Internet has made great contribution to mankind

The Internet can let me get the information I need

5. Internet makes society more progressive

**Part IV: ICT self-efficacy**

The following table is a self-assessment scale (18 questions) used to evaluate ICT self-efficacy. Please choose the one that best suits you for each of the following statements according to your situation.

1 = totally disagree, 2 = not agree, 3 = relatively agree, 4 = mostly agree, 5 = totally agree

1. I can easily hide any post shared / marked on my profile by someone on the social networking website I mainly use

2. I can easily block or restrict anyone on the social networking sites I mainly use

3. I can easily set a password on my mobile phone to ensure the security of my mobile phone.

4. I can easily change the password of my primary email / social network account.

5. I can easily unfriend anyone on the social networking sites I mainly use

6. I can easily disguise myself as me to report fake accounts.

7. I can easily report any ID, post, image or video as abuse / spam content on the social networking sites I mainly use

8. I can easily control the privacy settings of the social networking sites I mainly use

9. If I forget my password, I can easily recover my email / social network account.

10. I can easily deal with spam received by email or posted on the wall of social networking sites

11. I can easily judge whether the information provided by someone on social networking sites is correct.

12. I can easily judge the trusted information on social networking sites

13. I am fully aware of the consequences of my actions on the Internet.

14. I can easily express my views on any online forum.

15. When I open any website, I can easily learn how to use its functions in a very short time.

16. I can easily use chat rooms on the Internet.

17. I can easily talk to others through the webcam.

18. I can easily edit or modify any picture on my computer / mobile phone using different software

**Part V: Information Literacy of Higher Vocational Students**

The following table is a self-assessment scale (50 questions) for assessing information literacy. Please choose the one that best suits you for each of the following statements according to your situation.

1 = totally disagree, 2 = not agree, 3 = relatively agree, 4 = mostly agree, 5 = totally agree

1. Do you think that in modern life, it is important for us to timely grasp external information and understand the relevant national policies on the employment of Higher Vocational College Graduates

2. Which employers will you pay attention to will become internship units for higher vocational college graduates

3. As for the employment and probation system for graduates of higher vocational colleges that has been introduced, do you think it will help you find a job after graduation

Do you often look up information on the Internet?

5. How do you learn by using online resources (such as professional websites, online libraries, online knowledge education, etc.)

6. In the process of learning, you should choose software suitable for your major (such as access, dreamwave, Photoshop, flash, Authorware, 3ds, etc.):

7. Do you know the following concepts (such as: information theory, information retrieval, intellectual property, computer virus, information pollution, information crime, information source, Privacy)

8. In the process of learning, you will distinguish the use of the downloaded pictures, audio-visual materials, texts and other materials and apply them to the subject

9. When you get a lot of professional knowledge about the subject from the Internet, you will summarize and classify it

10. In the learning process, you can determine the type and scope of information required

11. On average, you spend one hour or more on the Internet every day

12. When you use computers to process words, pictures and audio-visual images, you can determine their types and ranges

13. You can successfully connect, install and use common input devices (such as mouse, keyboard, microphone, etc.) and output devices (such as monitor, printer, chassis, etc.)

14. Your search tools for some Internet information, such as "Yahoo", "Baidu", "Google", "3721", etc

15. You can use Internet Explorer to browse the web and download various articles, pictures, audio and video materials from the web

16. You can summarize suitable keywords and specific words, and develop good search strategies

17. When you enter different search systems to search for information, you can correctly understand its user interface and operation engine, and use different search methods to search for information

18. You can use various search tools such as (web browser and search engine tools, online library directories, magazines, newspaper indexes, etc.) to search various (such as voice, graphics, text materials, etc.) information

19. You can obtain the required information through online libraries, expert online, technical research institutions and other service websites

20. You can exchange knowledge with people all over the world through letters, surveys, interviews, e-mail, IRC real-time chat and other forms

21. You can determine the differences between the search contents and re formulate a search strategy

22. How do you download relevant network materials by using copy, paste, cut, save and other functions

23. You can store the downloaded materials in different folders according to different types

24. You can add and organize some frequently logged in websites into local or network favorites. For this, you can

25. You can use word software, image software, spreadsheet, etc. to process the downloaded data

26. You can summarize and synthesize the selected information and summarize the main ideas

27. You know how to maintain your computer, and you can use anti-virus software (such as rising, Norton, Kingsoft, etc.) to prevent and remove viruses from your computer on a regular basis

28. You will understand the background, culture and source of the downloaded materials in various ways, and analyze whether they are useful for learning

29. You can always actively learn new learning tools and explore new learning methods

30. Based on the collected information, you can synthesize the main ideas and form new solutions to problems in study and social life

31. You can compare the new knowledge with the previous knowledge to determine the added, contradictory or other characteristics of the new information

32. You will combine the new information with the original knowledge and select useful information from the acquired information

33. You will log in to some legal websites or use legal search engines to search and download information

34. Faced with the uneven quality of information resources, you can consciously resist and eliminate the interference and erosion of junk information and harmful information

35. What is your attitude towards the application of computers and networks in daily subject teaching

36. You can discuss and learn with your classmates, teachers, experts and others through e-mail, bulletin board (BBS), real-time chat and other information technology tools

37. You can judge whether the obtained information meets the requirements and adjust the search strategy

38. You can use knowledge theory and information tools to create various works (such as pictures, music, web pages, videos, etc.), for which you

39. You can combine the old and new information to convert the types of pictures, words, audio-visual and other files

40. Have you ever used blog or multimedia network to publish your own articles or technical works?

41. You often use various tools and software to process various information (pictures, words, audio-visual, etc.)

42. You can communicate and promote your work with others through BBS websites, QQ chat rooms, various technical websites, etc

43. You will distinguish between free and paid information on the Internet, taking into account its intellectual property rights and copyright

44. For communicating and transmitting information on the Internet, you should follow the ethical code and corresponding etiquette

45. When you log in to a website, you will register a legal account to log in and access information resources, and will abide by the systems and policies set by the website

46. You know about computer viruses and network security issues

47. You will obtain research results or articles published on the Internet by others through legal channels

48. In the Internet world of information, we should know how to respect other people's intellectual property rights and labor achievements

49. You can edit programs and design and make web pages

50. When you quote other people's copyrighted information, you will list the source and mark the quotation
